# Learning Science Beyond Sight: Conceptual Engagement of Elementary School Students with Visual Impairment through Hands-On Activities

**DOI:** 10.12688/f1000research.171534.1

**Published:** 2025-11-18

**Authors:** Resti Yektyastuti, Sarwanto, Rasmitadila

**Affiliations:** 1 Science Education, Universitas Sebelas Maret, Surakarta, Central Java, 57126, Indonesia; 2 Elementary School Teacher Education, Universitas Djuanda, Bogor, West Java, 16720, Indonesia

**Keywords:** Visual impairment, magnetism, conceptual understanding, science education, hands-on activities

## Abstract

**Background:**

A pressing challenge in science education is that visually impaired students continue to lack access to appropriate instructional media, making it difficult for them to engage with abstract concepts and resulting in limited opportunities for equitable education. Responding to this challenge, the present study investigates the design and implementation of hands-on, multisensory instructional strategies aimed at improving conceptual understanding of magnetism among elementary students with visual impairments.

**Method:**

The research involved four elementary school students with varying degrees of visual acuity. Employing the ADDIE (Analysis, Design, Development, Implementation, and Evaluation) framework, the research was divided into 3 stages: needs analysis (A) using explanatory case study, design and develop (DD) instructional media and validate using Aiken’s V, implement and evaluate (IE) the media into classroom activity using evaluative case study.

**Result:**

The findings indicate that tactile models, auditory explanations, and adapted activity kits significantly improved students’ engagement and comprehension of key magnetism concepts such as magnetic properties, forces, and polarity. Through an evaluative case study, the research demonstrates that instructional materials tailored to the sensory and cognitive profiles of visually impaired learners not only foster deeper conceptual understanding but also promote greater participation and confidence in science learning.

**Conclusions:**

Multisensory science kits help students access abstract science material so that it can be felt and helps achieve conceptual understanding. Moreover, the results may inform teachers, curriculum designers, and science education professionals on how to design and adapt science content in ways that are both accessible and pedagogically sound.

## Introduction

Science education is vital for fostering inquiry, critical thinking, and a fundamental understanding of the natural world ( Herwinarso et al., 2023; White & Delaney, 2021). For students with visual impairments, however, engaging meaningfully with science content poses distinct challenges due to the abstract science concept and often visually oriented nature of scientific concepts and materials ( Belay & Yihun, 2020; Kizilaslan et al., 2019). As a result, science instruction for students with visual impairments requires specialized approaches and materials tailored to their sensory and cognitive needs ( Kizllaslan & Sözbilir, 2020; Plazar et al., 2021).

Students with visual impairment face difficulties in lessons in which visual elements are predominant. Because they depend largely on visualization, science lessons are among the most difficult lessons for students in inclusive learning environments and their teachers, who need to find ways to accommodate their needs ( Kizilaslan, Zorluoglu, and Sozbilir, 2020; Ansari Ricci et al., 2019; Scholes and Stahl, 2020). However, science education can be made more accessible to students with visual impairments through specific adaptations in both classrooms and laboratories ( Greenvall et al., 2021; Koehler and Wild, 2019). In conventional science facilities, the lack of both tactile materials and hands-on science activities has been widely acknowledged as a major barrier to visually impaired students’ full participation in science lessons and ability to develop scientific thinking skills ( Rule et al., 2011; Tanwar, 2019). These students’ science learning concepts can be facilitated with materials that emphasize the sense of touch ( Ansari Ricci et al., 2019; Wild, 2011).

Within the framework of special education, instruction for students with visual impairments must emphasize non-visual modes of learning, such as tactile, auditory, and kinesthetic strategies. These approaches are not merely compensatory, but are essential pedagogical tools for helping students construct mental models of scientific phenomena that they cannot observe directly ( Kizllaslan & Sözbilir, 2020; Kızılaslan & Zorluoğlu, 2019; Langdon, 2020). The development of adapted learning materials and tactile science kits has shown promise in supporting the conceptual understanding of topics such as electricity, magnetism, and matter ( Herwinarso et al., 2023; Kapucu & Kızılaslan, 2022; Senjam et al., 2020; Widiyawati & Nurwahidah, 2018).

Magnetism is a core topic in elementary science that introduces students to invisible forces shaping both natural phenomena and everyday technology. For students with visual impairments, learning about magnetism holds particular importance, as it offers opportunities to explore abstract scientific ideas through concrete, tactile, and experiential activities. Rather than relying solely on visual demonstrations, concepts such as magnetic force, attraction and repulsion, and magnetic poles can be transformed into meaningful learning experiences through hands-on experimentation and adapted instructional tools. Beyond classroom learning, understanding magnetism also contributes to daily independence—for example, through the use of compasses for orientation, magnetic closures in household objects, and technologies embedded in assistive devices. Such approaches not only make magnetism accessible but also strengthen students’ abilities to reason about cause-and-effect relationships in science. Prior studies have demonstrated that when appropriate modifications are made, students with visual impairments can achieve a level of conceptual understanding comparable to their sighted peers. ( Iyamuremye et al., 2023; Widiyawati & Nurwahidah, 2018).

While magnetism illustrates these specific obstacles, it also underscores the urgent need for broader instructional adaptations across science subjects **.** Science concepts are often abstract and heavily rely on visual representations, which poses significant barriers for students with visual impairments. The case of magnetism highlights how invisible forces and spatial relationships, typically conveyed through visual demonstrations, represent a wider challenge in science learning. Without meaningful non-visual access to such content, these students may struggle to construct accurate and functional conceptual understandings of key scientific principles ( Gustiani et al., 2022; Lahav et al., 2022; Winters et al., 2020).

Furthermore, based on previous studies, science instruction for students with visual impairments should incorporate multisensory and contextually grounded learning experiences ( Bandyopadhyay & Rathod, 2017; Gustiani et al., 2022; Junilasari et al., 2019). Multisensory learning allows students to access scientific concepts through tactile exploration ( Senjam et al., 2020), auditory explanations ( Lahav et al., 2016), and hands-on activities ( Kizilaslan et al., 2019). When these experiences are embedded within meaningful, real-life contexts—what is known as contextual learning—students are more likely to relate scientific concepts to everyday experiences, leading to deeper understanding and long-term retention ( Lotlikar et al., 2020).

Contextual science learning emphasizes learning through concrete experiences and authentic tasks. For learners with visual impairments, this might involve manipulating real objects, participating in simplified experiments, or engaging in guided exploration using adapted tools and materials ( Fernández et al., 2019). Through such approaches, abstract concepts become tangible and comprehensible. In the case of magnetism topic, for example, students can directly manipulate magnets and various materials to explore properties like attraction and repulsion, polarity, and magnetic force—thus constructing mental models grounded in personal, active engagement rather than passive verbal instruction alone.

However, there is still limited research that systematically explores the process of designing, implementing, and evaluating instructional materials specifically intended for students with visual impairment in the context of elementary science education ( Ediyanto & Kawai, 2019; Yektyastuti et al., 2024). This study aims to investigate the development of tailored instructional activities and materials for teaching the concept of magnetism to elementary students with visual impairments.

The findings are expected to provide insights into how science learning experiences for students with visual impairment can be designed to promote active engagement, conceptual development, and meaningful application of scientific knowledge through contextually rich, multisensory strategies.

## Method

### Participants

The study involved four elementary students with visual impairments (grades 5) selected through purposive sampling, as presented in Table 1. The inclusion criteria were: (1) formally registered in the special needs school, (2) diagnosed with low vision or total blindness, and (3) having basic literacy in Braille or auditory learning. The exclusion criteria were students with multiple disabilities that could significantly affect participation in science learning. In addition, two teachers were included in the study: a non-disabled science teacher and a special education teacher (totally blind). The same group of students and teachers continued to participate in the subsequent development and implementation stages to maintain coherence and ensure comparability of findings.

**Table 1.** Students as sample.

Student	Gender	Age	Visual acuity
S _1_	Female	10	Totally Blind (congenital, no light perception)
S _2_	Female	11	Low Vision (limited central vision, uses magnifier)
S _3_	Male	11	Totally Blind (congenital, no light perception)
S _4_	Male	10	Low Vision (residual vision, uses magnifier)

Prior to the commencement of the study, both students and teachers who were involved as participants provided their informed consent to voluntarily participate in the research. Their participation was entirely voluntary, free from any form of coercion or undue influence. For the student participants, consent was obtained through their teachers and parents, as documented in the official consent letter number 076/III/012-Suket/2025.

### Ethical consideration

Before the research session, the objectives of the study were clearly explained, and respondents’ consent for data collection was obtained. All respondents voluntarily agreed to participate without any form of coercion or undue influence. Informed consent was obtained from both schools and students. For student participants with visual impairments, informed consent was given verbally due to their visual limitations, and this verbal consent was subsequently documented in writing by their teachers. The written record of consent was formalized through an official consent letter (No. 076/III/012-Suket/2025). Furthermore, all research procedures and data collection plans were communicated to the Research and Community Service Institute of Universitas Djuanda and received ethical approval through official letter number: 001/KEP/LPPM/III/2025. To ensure confidentiality and data security, participants’ identities were anonymized, and all interview and observation records were securely stored.

### Procedure

**Figure 1.**
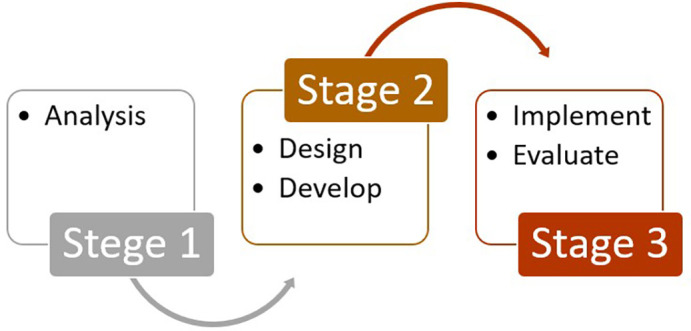
Research stages.

This study employed the ADDIE (Analysis, Design, Develop, Implement, and Evaluate) as the methodological framework ( Branch, 2009). Unlike previous studies that primarily applied ADDIE for instructional product development, this research emphasized the implementation and evaluation phases to test the effectiveness of multisensory instructional strategies for students with visual impairments. By combining exploratory and evaluative case study approaches, the design enabled a systematic process of identifying needs, developing materials, validating products, and assessing outcomes in real classroom contexts. A flowchart of the research procedure is presented in Figure 1.

**In the first stage** ( **analysis**), an exploratory case study was conducted to identify student needs related to the physical environment, teaching methods, learning activities, and evaluation approaches ( Branch, 2009; Schott & Seel, 2015). Data were collected in April 2025 through classroom observations and semi-structured interviews with teachers and students. Observation protocols included parameters such as classroom interaction, accessibility of materials, and student engagement. Interviews were guided by semi-structured protocols focusing on learning barriers and needs. Instruments used included observation sheets, interview guides, and audio recorders.

**In the second stage (design and develop),** learning activities and supporting materials were developed based on the findings of the first stage. These included a multisensory science kit, teacher guides, student worksheets, and practice materials focused on magnetism concepts and learning outcomes as presented in Table 2.

**Table 2.** Learning outcomes.

Topic	Magnetism
**Level**	5 ^th^ grade
**Learning outcome**	Students understand that force can affect the motion of objects, as well as identify the properties of magnets and their uses
**Learning objectives**	Students are able to describe the properties of magnet
	Students are able to conduct experiments to identify the properties of magnets
	Students are able to report the results of experiments related to the properties of magnets

The initial product was then validated by experts in science education, special education, and educational technology. Prior to validation, preliminary discussions with the experts were conducted to establish shared expectations and criteria. The validation process employed a structured questionnaire. The results were analyzed using Aiken’s V equation ( Aiken, 1985; Shultz et al., 2021) with 4 item categories and 6 raters. The calculated Aiken’s V was then compared to the critical value in the Aiken’s V table (0.78).

The validation of the magnetism science activity kit was conducted through expert judgment involving six experts, consisting of two in science education, two in special education, and two in educational technology. The evaluation instrument comprised 13 items, each rated on a 4-point scale (1 = not relevant, 4 = highly relevant). To determine the validity of each item, Aiken’s V coefficient was applied. Aiken’s V is calculated per item based on the ratings provided by all experts, using the following formula:

$$V=\frac{\mathrm{\Sigma s}}{n(c-1)}$$

where s = r−ls = r - ls = r−l. In this formula, r represents the score assigned by an expert, l denotes the lowest possible score on the rating scale, n is the total number of experts involved in the validation process, and c refers to the number of categories in the rating scale.

Aiken’s V values range from 0 to 1, where values closer to 1 indicate stronger agreement among experts regarding the relevance of the item. In this study, each of the 13 items was evaluated by all six experts, producing an item-level validity coefficient. The overall validity of the instrument was then determined by calculating the average of these item-level coefficients.

Data collection for this stage took place from May to June 2025. Expert feedback was also used for iterative revisions, and improvements were made until the product was deemed valid and appropriate for classroom use.

**The third stage (implement and evaluate)** focused on testing the validated kit in a grade 5 magnetism unit at a special needs school. Units of study included magnetic properties, forces, and polarity. The evaluative case study method was used to assess students’ conceptual understanding, combining detailed description, explanation, assessment, and causal analysis ( Smeyers & Depaepe, 2019). The participants in this stage were students, the same as those involved in the first stage to ensure continuity and consistency of data.

Data were collected in July 2025 through pre- and post-tests, classroom observations, and performance tasks. Instruments included test sheets, observation rubrics, and audio recordings. Data were analyzed quantitatively using descriptive statistics to measure learning gains, and qualitatively using thematic analysis of student responses and classroom interactions. A summary of the stages, data collection methods, instruments, and types of data is presented in Table 3.

**Table 3.** Summary of the method.

Research stage	Type of data	Data collection method	Instruments	Data analysis
**Analysis**	Student needs (learning barriers, accessibility, participation); classroom environment	Classroom observation; Semi-structured interview with teachers and students	Observation sheets, interview guides, audio recorder	Thematic analysis using NVivo to identify categories
**Design & Development**	Draft of multisensory science kit and supporting materials; Expert judgments and feedback	Expert discussion and validation	Validation questionnaire, expert review notes	Aiken’s V formula to determine validity indices; qualitative synthesis of expert comments for revisions
**Implementation & Evaluation**	Student conceptual understanding of magnetism (magnetic properties, forces, polarity); Student engagement	Pre- and post-tests; Classroom observation; Performance tasks	Test sheets, observation rubrics, audio recordings	Descriptive statistics for learning gains, student responses, and classroom interactions

## Results

### Stage 1. Analysis

An exploratory case study was conducted by observing science learning for students with visual impairments, followed by interviews. The observations revealed that science lessons were conducted in a combined class that included students from grades 1 to 6. Although the grade levels differed, all students learned the same topic simultaneously. During instruction, the teacher provided verbal explanations and guided students to write and answer questions using Braille tools such as reglet and stylus. After completing the tasks, students read their Braille writing aloud, allowing the teacher to check their understanding. This learning pattern was consistently implemented across subjects, including science, and reflects a strong emphasis on verbal interaction and tactile engagement as primary learning modalities.

**Figure 2.**
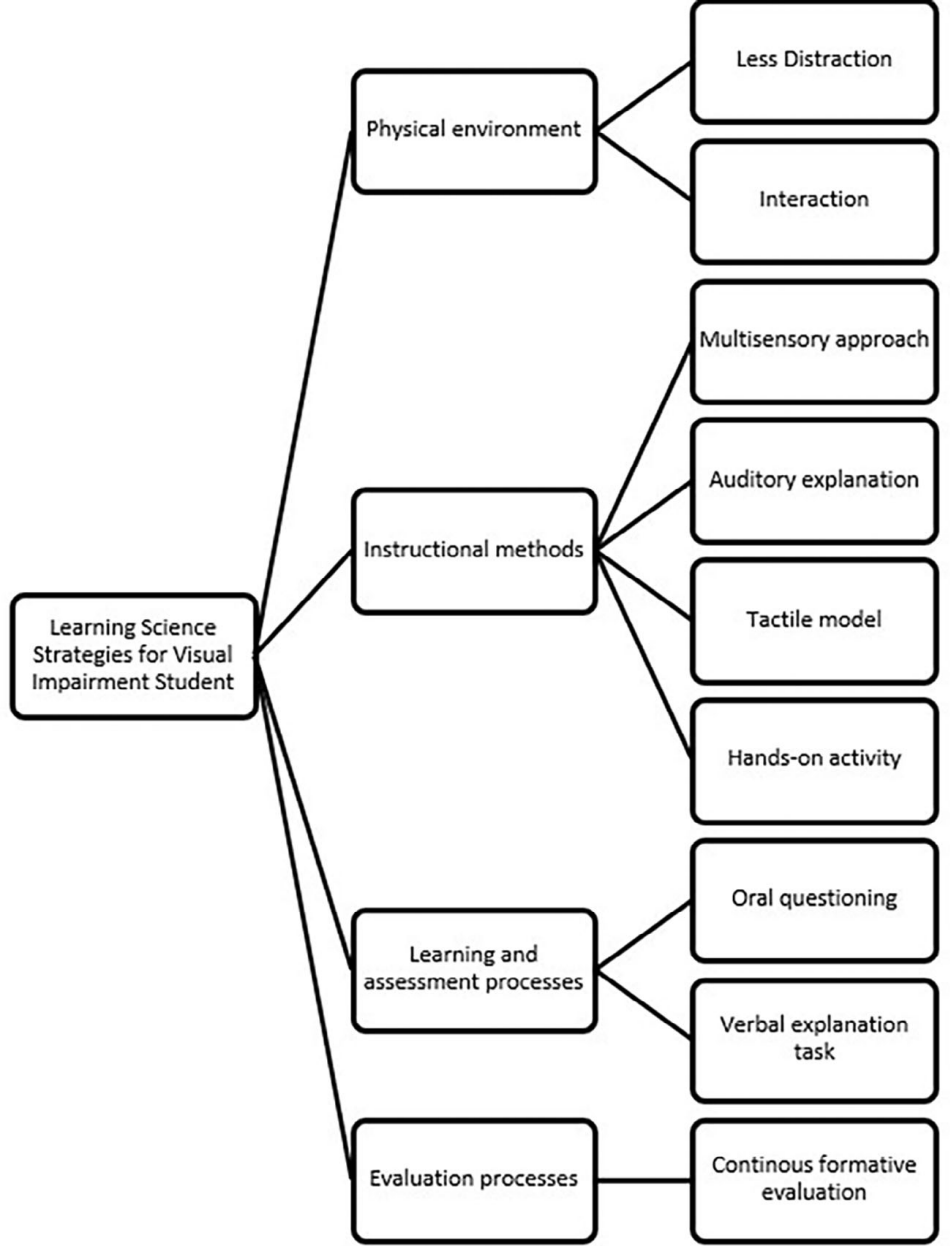
Thematic analysis result.

The results of the first phase of the exploratory case study ( Yektyastuti, 2025a) revealed several important needs of students with visual impairments in science learning, particularly in the topic of magnetism. These needs were grouped into four main categories as presented in Figure 2, namely: 1) Physical environment: Students require a learning space with minimal visual distractions, tactile cues, and seating arrangements that facilitate interaction with both the learning materials and the teacher; 2) Instructional methods: Students prefer a multisensory approach, including auditory explanations, tactile models, and hands-on experiments. Verbal explanations alone are insufficient for conceptual understanding; 3) Learning and assessment processes: Assessments should include oral questioning, tactile identification, and verbal explanation tasks; and 4) Evaluation processes: Continuous formative evaluations provide a better picture of student understanding and allow for adjustments to learning. These findings support previous research emphasizing the need for adaptive instructional strategies for students with visual impairments.

Based on these identified needs, the product design of the science kit was directed to include tactile accessible magnet models, various magnetic and non-magnetic objects, and instructional materials available in multiple formats (print, braille, and audio). This ensured that the developed kit was not only aligned with curricular goals but also responsive to the sensory and cognitive profiles of the learners.

### Stage 2. Design and develop

**Figure 3.**
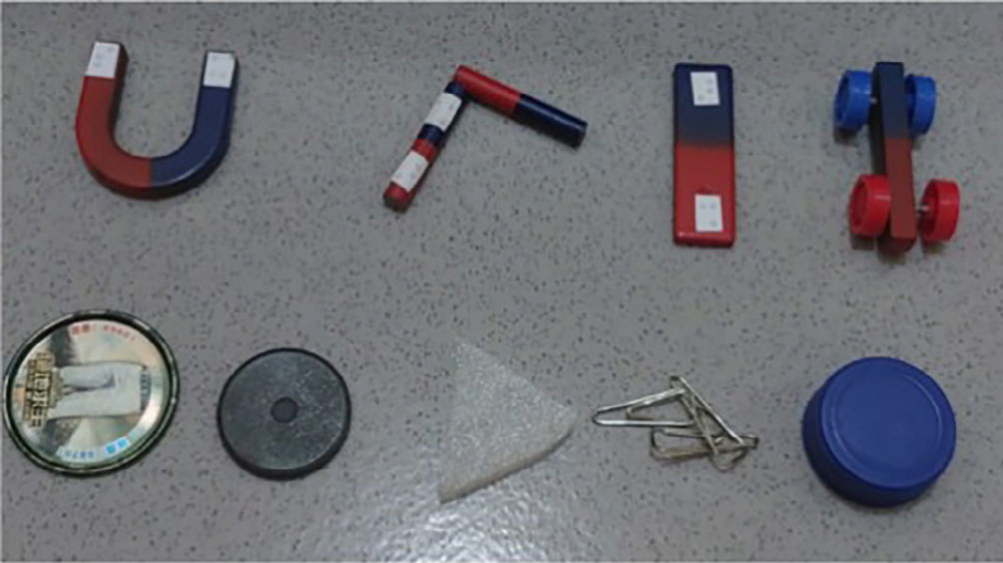
Tactile magnetic kit.

The results of the analysis in the first phase formed the basis for the development of the second phase product. The product was a science activity kit for magnetism. The product was designed to achieve learning objectives in accordance with the applicable curriculum for special education elementary schools in Indonesia. The science activity kit for magnetism consists of several components: magnets in various models, magnetic objects, and non-magnetic objects. The magnetism kit components are shown in Figure 3. The magnets are tactilely marked to indicate the north and south poles. The kit also includes an activity guidebook accessible in written, braille, and audio formats.

The developed magnetism science activity kit was reviewed by six experts: two in science education, two in special education, and two in educational technology. Validation was conducted using a 13-item instrument rated on a 4-point scale. Aiken’s V coefficient was calculated for each item, with values above 0.80 indicating strong validity. Table 4 summarized the validation results of the initial product ( Yektyastuti, 2025b).

**Table 4.** Expert judgment.

Aspect	Indicators	Expert						Σs	Aiken’s V	Criteria
		1	2	3	4	5	6			
Accuracy of subject material	Accordance with curriculum	4	4	3	3	3	4	15	0.833	Valid
	Accordance with applicable scientific principles	4	4	3	3	3	4	15	0.833	Valid
	Engagement of science concept	4	4	3	3	3	4	15	0.833	Valid
Compliance with User Needs	Science KITs are accessible to visually impaired students	4	4	3	3	3	4	15	0.833	Valid
	The guidebook in the KIT	4	4	3	3	3	4	15	0.833	Valid
Function and Effectiveness	Support students’ understanding of science concepts	4	4	3	3	3	4	15	0.833	Valid
	Support students practice science process skills	4	4	3	3	3	4	15	0.833	Valid
Practicality	KIT is easy to use	4	4	3	3	3	4	15	0.833	Valid
	Equipped with all the necessary components	4	4	3	3	3	4	15	0.833	Valid
Security	Safe for students to use	4	4	3	3	3	4	15	0.833	Valid
	Durable and not easily damaged	4	4	3	3	3	4	15	0.833	Valid
Aesthetics	Attractive design and support student learning	4	4	4	4	3	3	16	0.889	Valid
	suited to the needs of visually impaired students	4	4	4	4	3	3	16	0.889	Valid
**Average**									**0**. **842**	

The magnet kit was then implemented in the classroom science lesson. The teacher provided all students with a verbal introduction to the topic of magnet. The students were then given an explanation of the learning steps using the magnet kit. They observed the magnet kit by touch, read the tactile activity guide, and then carried out learning activities according to the guide.

Recommendations summary from experts:

• In addition to the magnetic and nonmagnetic objects provided in the kit, encourage students to explore objects around them. Then, ask them to classify their magnetic properties.
• Add a box that integrates all the kit components, including the instruction manual. Provide braille markings on the box and each component.

Based on expert recommendations, several improvements were made to enhance the functionality and accessibility of the magnetism science activity kit. The activity guide was expanded to encourage students to explore and classify everyday objects by their magnetic properties, fostering independent and contextual learning. Additionally, a storage box was designed to integrate all kit components, with braille markings on both the box and each item to ensure accessibility and ease of use for students with visual impairments.

### Stage 3. Implement and evaluate

The magnet kit was then implemented in learning through 2 science activities. In **Activity 1**, students identified magnetic poles. Two different magnets were observed by touch to identify their pole types. The poles of the two magnets were brought close together to observe the repulsion and attraction properties of magnets. Like poles repel, while unlike poles attract. Students experienced the repulsion and attraction between the poles. The activity then continued with one magnet with pole markers and another magnet without markers. Students were then asked to identify the pole type of the unmarked magnet based on the magnetic force it generated when brought close to the magnet with the marked poles. In **Activity 2**, students identified magnetic and nonmagnetic objects provided in the kit and objects around them. Based on the magnetic force generated when an object is brought close to a magnet, students can identify whether the object is magnetic or nonmagnetic. The students then present their observations from all of these activities verbally. The teacher administers a verbal test to each student to determine their conceptual understanding of magnets.

In the final stage, an evaluative case study was conducted to assess the effectiveness of the designed materials and activities. Table 5 presents the pre- and post-test results of student understanding of magnetism ( Yektyastuti, 2025c). The average pre-test score was 3.5, which increased to 8.25 in the post-test, with an average gain of +4.75 across the four participants. This improvement demonstrates substantial learning progress following the implementation of the adapted instructional materials. The four participating students (two blind and two with low vision) showed notable improvement in their understanding of magnetism concepts. Students with blindness (S _1_ and S _3_) were able to identify magnetic materials, explain the presence of magnetic forces, and demonstrate understanding of magnetic poles using tactile models and oral explanations. Students with low vision (S _2_ and S _4_) benefited from a combination of visual enlargement and tactile materials, showing comparable gains in concept mastery.

**Table 5.** Result of evaluation.

Student	Visual acuity	Pre-test score	Post-test score	Gain	Description of conceptual understanding
S _1_	Blind	3	8	+5	Able to identify magnetic materials, explain the presence of magnetic forces, and demonstrate understanding of magnetic poles using tactile models and oral explanations
S _2_	Low Vision	4	8	+4	Get benefit from a combination of visual enlargement and tactile materials. Able to identify magnetic materials, explain the presence of magnetic forces, and demonstrate understanding of magnetic poles using tactile models and oral explanations
S _3_	Blind	2	7	+5	Able to identify magnetic materials, explain the presence of magnetic forces, and demonstrate understanding of magnetic poles using tactile models and oral explanations
S _4_	Low Vision	5	9	+4	Get benefit from a combination of visual enlargement and tactile materials. Able to identify magnetic materials, explain the presence of magnetic forces, and demonstrate understanding of magnetic poles using tactile models and oral explanations

The evaluation highlighted that when instruction was adapted to students’ sensory modalities, all participants were able to engage meaningfully with scientific content. This finding aligns with ( Smeyers & Depaepe, 2019) assertion that evaluative case studies can effectively reveal causal links between instructional interventions and learning outcomes. Additionally, students reported increased confidence and enjoyment during science lessons. Teachers also noted greater student participation and engagement compared to previous lessons without adapted materials. Taken together, the quantitative evidence of learning gains and the qualitative insights from observations and interviews reinforce each other, showing not only measurable improvements in test performance but also richer engagement, confidence, and independence in science learning among visually impaired students.

## Discussion

The findings of this study underscore the critical importance of designing science instruction that is responsive to the unique sensory, cognitive, and experiential needs of students with visual impairments. Common science education often assumes visual access to phenomena, materials, and representations, which unintentionally marginalizes learners who process information through non-visual modalities. This study demonstrates that when instructional practices and materials are thoughtfully adapted—using tactile, auditory, and kinesthetic strategies—students with visual impairments are capable of developing a robust conceptual understanding of abstract scientific concepts, such as magnetism. This finding underscores that conceptual barriers in science are not inherent to disability itself, but rather to the lack of accessible instructional design. These results align with prior research indicating that the use of multisensory approaches not only supports access but also enhances engagement and learning outcomes among students with disabilities ( Bandyopadhyay & Rathod, 2017; Fernández et al., 2019; Gustiani et al., 2022; Kizilaslan et al., 2019).

One of the central contributions of this study is the emphasis on systematic instructional design grounded in the analysis of student needs. The initial phase of the research involved an exploratory case study that identified critical barriers and instructional gaps experienced by students with visual impairments in science classrooms. This foundational step ensured that the instructional materials and learning activities developed in the second phase were not only accessible but also pedagogically aligned with how visually impaired learners process and internalize scientific content. This approach reflects the principles of Universal Design for Learning (UDL) ( Langdon, 2020; Meyer et al., 2014; Tosun, 2022), which advocate for flexible, learner-centered instruction that anticipates variability in learners’ abilities, experiences, and backgrounds. The materials designed in this study—particularly the tactile learning aids and adapted activity guides—exemplify how inclusive instructional design can be achieved through an iterative and evidence-informed process.

Building on these design principles and the needs identified in the exploratory phase, the development of the science kit was directed to integrate tactile and auditory features that make abstract concepts of magnetism more tangible. The kit included three main components: (a) tactilely accessible magnet models with distinct shapes and sizes that enable students to explore magnetic poles and forces through touch; (b) a set of magnetic and non-magnetic objects made of different materials, allowing learners to conduct classification activities and hands-on experiments; and (c) instructional guides and worksheets provided in multiple formats, including print, braille, and audio recordings, ensuring accessibility for diverse sensory preferences ( Bandyopadhyay & Rathod, 2017; Fernández et al., 2019; Saleem & Al-Salahat, 2016). In addition, the kit was designed with safety considerations and durable materials suitable for repeated classroom use. By aligning its components with the identified learning needs, the science kit served not only as a tool for instruction but also as a medium to foster independent exploration, meaningful interaction, and equitable participation in science learning for students with visual impairments ( Fernández et al., 2019; Kizilaslan et al., 2019, 2020). This intentional alignment illustrates how adapted resources can move beyond accommodation toward genuine empowerment, enabling students to participate as active constructors of scientific knowledge rather than passive recipients. From a theoretical standpoint, this approach is grounded in constructivist learning theory ( Carey et al., 1989; Milton, 1976), which emphasizes knowledge construction through active engagement and personal experience, and is further supported by the principles of multisensory learning, where the integration of tactile, auditory, and kinesthetic modalities enhances comprehension and retention of abstract concepts.

Furthermore, the evaluative case study methodology used in the third phase of this research provided valuable insights into the impact of the instructional intervention on students’ conceptual understanding. Through qualitative observations and performance-based assessments, the study captured the depth of students’ engagement with the materials and their ability to articulate scientific concepts using appropriate reasoning.

Importantly, this study contributes to the expanding literature on inclusive science education by offering both empirical evidence and a practical model for the design, implementation, and evaluation of adapted science instruction for students with visual impairments. While much of the existing research focuses on general inclusion practices or high-level policy frameworks, this study provides concrete instructional strategies and materials that can be replicated or adapted by teachers and curriculum developers. It also responds to a notable gap in the literature—that focus on specific content areas (such as magnetism) and examine learning outcomes in depth ( Akarsu et al., 2021; Kizilaslan et al., 2019). Few prior studies have systematically connected the process of needs analysis, iterative kit design, and evaluative testing in one research cycle. This study therefore contributes a methodological template for future design-based research in special education.

Moreover, the implementation of such adapted instructional materials within inclusive classrooms can be further strengthened through the active role of supporting teachers (special education teachers or teaching assistants). Their presence enables individualized scaffolding, ensures that adaptations are effectively integrated into classroom routines, and facilitates collaboration with general education teachers to create an equitable learning environment for both visually impaired students and their sighted peers ( Rasmitadila et al., 2020; Rasmitadila & Goldstein, 2017).

The results of this study affirm several key implications. Instruction tailored to sensory and cognitive profiles of students with visual impairments can significantly improve access to, and understanding of, abstract scientific content. The development of inclusive learning materials must begin with a thorough analysis of learner needs, ensuring that adaptations are not merely compensatory, but transformational. Evaluative case studies offer a robust methodology for examining the educational impact of instructional innovations in specialized contexts, particularly when quantitative measures are insufficient. For teachers, this study offers a replicable model of lesson design that combines tactile experimentation with guided inquiry. For policymakers and curriculum developers, it highlights the need to embed accessibility considerations from the outset of curricular planning rather than as afterthoughts.

This research provides a scalable model for inclusive instructional design that may inform broader efforts to make science education more accessible, equitable, and effective for all learners, including those with disabilities. By integrating multisensory, contextual, and inquiry-based strategies, this study not only expands opportunities for students with visual impairments to engage with science meaningfully, but also challenges conventional notions of how scientific knowledge can be constructed and demonstrated in diverse learning environments.

## Conclusions

This study confirms that thoughtfully adapted instructional practices, grounded in the sensory and cognitive needs of students with visual impairments, can significantly enhance their conceptual understanding of abstract science topics such as magnetism. By integrating tactile, auditory, and hands-on learning strategies, students were able to meaningfully engage with scientific phenomena that are typically conveyed through visual demonstrations.

The findings emphasize the value of conducting thorough needs assessments prior to material development, ensuring that adaptations are pedagogically sound and inclusive rather than merely compensatory. Furthermore, the evaluative case study approach provided rich insights into individual learning progress and instructional effectiveness.

In line with prior research on multisensory learning for students with disabilities, this study extends the evidence base by demonstrating how structured instructional kits can be systematically designed and validated for use in special education settings. In doing so, it successfully addresses the research gap identified in the introduction, namely the limited availability of empirically tested science instructional models tailored for learners with visual impairments.

The results also indicate that the study objectives—identifying instructional needs, developing and validating a magnetism activity kit, and evaluating its effectiveness—were successfully fulfilled.

Beyond the local context, the findings carry broader implications for global discussions on inclusive science education. As education systems worldwide move toward equity and universal access, this study illustrates how science learning can be reimagined beyond visual modalities, offering a replicable model that can inform practices in diverse cultural and educational contexts. Ultimately, this research contributes to expanding educational opportunities for learners with disabilities and advancing more equitable, multisensory approaches to science education.

### Limitations

The study involved a small number of participants (four students and two teachers) from a single special needs school, which limits the generalizability of the results. The focus on a single science topic—magnetism—means that further research is required to examine whether similar approaches are effective for other abstract science concepts. In addition, the evaluative case study design provided in-depth insights but did not allow for comparison with a control group. Future research should consider larger samples, diverse school contexts, and comparative designs to strengthen the external validity of these findings.

## Data Availability

• Figshare. Instrument and data from Observation and Interview. DOI: http://doi.org/10.6084/m9.figshare.30231700 ( Yektyastuti, 2025a) • Figshare. Instrument and data from expert judgment. DOI: http://doi.org/10.6084/m9.figshare.30163915 ( Yektyastuti, 2025b) • Figshare. Instrument and data from test of conceptual understanding. DOI: http://doi.org/10.6084/m9.figshare.30254701 ( Yektyastuti, 2025c) Data are available under the terms of the Creative Commons Attribution 4.0 International license (CC-BY 4.0).
