# Supplementary material for: Learning Science Beyond Sight: Conceptual Engagement of Elementary School Students with Visual Impairment through Hands-On Activities
Source: F1000Res. 2026 Jan 8;14:1271. Originally published 2025 Nov 18. [Version 2] doi: 10.12688/f1000research.171534.2 (PMC12810695; doi:10.12688/f1000research.171534.2)
Supplement: Peer review history

# Reviewer report: Reviewer response for version 2

**DOI:** 10.5256/f1000research.194748.r449049

**Published:** 2026-01-16

**Authors:** Afriyanti Afriyanti

**Affiliations:** 1 Universitas Gunung Kidul, Gunung Kidul Regency, Special Region of Yogyakarta, Indonesia

The research topic addresses the challenge that education must be equitable, including for students with visual impairments. The multisensory learning tools developed by the researchers will greatly assist teachers in teaching science to students who are visually impaired. 

 The study design appropriate and is the work technically sound. all the source data underlying the results available to ensure full reproducibility. the conclusions drawn adequately supported by the results.

Is the work clearly and accurately presented and does it cite the current literature?

Yes

If applicable, is the statistical analysis and its interpretation appropriate?

Yes

Are all the source data underlying the results available to ensure full reproducibility?

Yes

Is the study design appropriate and is the work technically sound?

Yes

Are the conclusions drawn adequately supported by the results?

Yes

Are sufficient details of methods and analysis provided to allow replication by others?

Yes

Reviewer Expertise:

My expertise is in the field of science education. I have developed a science learning model, particularly in physics, namely the OPEK learning model. Another area of my expertise is quantitative analysis based on the Rasch model.

I confirm that I have read this submission and believe that I have an appropriate level of expertise to confirm that it is of an acceptable scientific standard.

## References

[1] : Science for Students with Visual Impairment: An Analysis of Hands-on Activity. *Bartın University Journal of Faculty of Education* .2021;2021 February, Volume 10(Issue 1) : 10.14686/buefad.627796 51-68 10.14686/buefad.627796

[2] : Examining the use of adaptive technologies to increase the hands-on participation of students with blindness or low vision in secondary-school chemistry and physics. *Chemistry Education Research and Practice* .2016;17(4) : 10.1039/C6RP00141F 1174-1189 10.1039/C6RP00141F

# Reviewer report: Reviewer response for version 2

**DOI:** 10.5256/f1000research.194748.r448547

**Published:** 2026-01-12

**Authors:** Siti Masyithoh

**Affiliations:** 1 Universitas Islam Negeri Syarif Hidayatullah Jakarta, South Tangerang, Banten, Indonesia

Dear Authors,

 Thank you for your revision to increase the quality of the manuscript. Your revisions are very detail and suitable for the indexing.

Is the work clearly and accurately presented and does it cite the current literature?

Yes

If applicable, is the statistical analysis and its interpretation appropriate?

Yes

Are all the source data underlying the results available to ensure full reproducibility?

Partly

Is the study design appropriate and is the work technically sound?

Yes

Are the conclusions drawn adequately supported by the results?

Yes

Are sufficient details of methods and analysis provided to allow replication by others?

Partly

Reviewer Expertise:

Character and inclusive education in elementary schools

I confirm that I have read this submission and believe that I have an appropriate level of expertise to confirm that it is of an acceptable scientific standard.

# Reviewer report: Reviewer response for version 1

**DOI:** 10.5256/f1000research.189151.r434010

**Published:** 2025-12-30

**Authors:** Minsih Minsih

**Affiliations:** 1 Universitas Muhammadiyah Surakarta, Surakarta, Indonesia

A relevant and well-structured study with clear practical value. The main issues relate to clarity of reporting, not methodology. Minor revisions will strengthen the narrative and improve transparency. The paper clearly and accurately presented and cite the current literature.The study design appropriate and is the work technically sound. The statistical analysis and its interpretation appropriate. The analysis of the discussion is in-depth and relevant to the correct citation system. The novelty in the introduction is clearly visible. Improvements that need to be made to this paper are in the introduction: The problem is well presented, but the manuscript needs a more explicit identification of the gap in existing literature. The theoretical basis for multisensory learning should appear earlier. The ADDIE process is described well, but the study lacks detail on how the pre–post tests were adapted for blind and low-vision students. Clarify delivery format (braille/audio/oral) and how the adapted assessments were validated.

Is the work clearly and accurately presented and does it cite the current literature?

Yes

If applicable, is the statistical analysis and its interpretation appropriate?

Yes

Are all the source data underlying the results available to ensure full reproducibility?

Yes

Is the study design appropriate and is the work technically sound?

Yes

Are the conclusions drawn adequately supported by the results?

Yes

Are sufficient details of methods and analysis provided to allow replication by others?

Yes

Reviewer Expertise:

A relevant and well-structured study with clear practical value. The main issues relate to clarity of reporting, not methodology.

I confirm that I have read this submission and believe that I have an appropriate level of expertise to confirm that it is of an acceptable scientific standard.

# Response

**Published:** 2026-01-01

**Authors:** Resti Yektyastuti

**Affiliations:** Department of Education, Universitas Djuanda, Bogor, West Java, Indonesia

Thank you very much for your thoughtful review and positive assessment of our manuscript. We sincerely appreciate your constructive comments, which have helped improve the clarity and transparency of the paper. We are grateful for your recommendation for acceptance and for the time and expertise you devoted to reviewing this work.

# Reviewer report: Reviewer response for version 1

**DOI:** 10.5256/f1000research.189151.r434009

**Published:** 2025-12-24

**Authors:** Siti Masyithoh

**Affiliations:** 1 Universitas Islam Negeri Syarif Hidayatullah Jakarta, South Tangerang, Banten, Indonesia

**MANDATOR**Y revisions:

 1. Add a minimally detailed explanation of the instrument and data analysis.

 2. Ensure source data is available (grades, rubric, Aiken's V table, etc.).

 3. Add a limitations paragraph.

 4. Improve consistency of citations and references.

**RECOMMENDED** revisions:

 1. Add an ADDIE flowchart to strengthen the visualization of the process.

 2. Add sample interview quotes to enrich the qualitative findings.

Is the work clearly and accurately presented and does it cite the current literature?

Yes

If applicable, is the statistical analysis and its interpretation appropriate?

Yes

Are all the source data underlying the results available to ensure full reproducibility?

Partly

Is the study design appropriate and is the work technically sound?

Yes

Are the conclusions drawn adequately supported by the results?

Yes

Are sufficient details of methods and analysis provided to allow replication by others?

Partly

Reviewer Expertise:

Character and inclusive education in elementary schools

I confirm that I have read this submission and believe that I have an appropriate level of expertise to confirm that it is of an acceptable scientific standard, however I have significant reservations, as outlined above.

# Response

**Published:** 2026-01-02

**Authors:** Resti Yektyastuti

**Affiliations:** Department of Education, Universitas Djuanda, Bogor, West Java, Indonesia

We sincerely thank you for the constructive comments and helpful suggestions provided to improve the clarity and transparency of our manuscript. We have carefully addressed all mandatory and recommended revisions as outlined below.

1. A clearer and more detailed explanation of the research instruments and data analysis procedures has been added to the *Procedure* section. This includes explicit descriptions of the assessment instruments, expert validation using Aiken’s V, and the quantitative and qualitative analysis approaches applied at each stage of the ADDIE framework.
2. All relevant source data, including student scores, observation rubrics, expert validation results, and the Aiken’s V table, are now made available in the *Data Availability* section at the end of the article, in line with the journal’s open data policy.
3. A dedicated *Limitations* section has been included after the conclusion, explicitly discussing the sample size, research context, topic scope, and design constraints of the study.
4. The manuscript has been carefully reviewed to ensure consistency and accuracy of citations and references throughout the text, following the journal’s referencing guidelines.
5. An ADDIE-based research flowchart has been included as *Figure 1*, visually summarizing the research stages and strengthening the methodological presentation.
6. Representative interview excerpts from teachers and students have been incorporated into the *Findings (Stage 1 Analysis)* section. These quotations directly support the thematic analysis and enrich the qualitative interpretation of the results.

Once again, we thank you for the insightful feedback, which has significantly strengthened the quality, clarity, and transparency of this manuscript.
